# Trends and Sociodemographic Differentials in Neonatal, Infant, and Under‐5 Mortality in Bangladesh, 1993–2022

**DOI:** 10.1002/puh2.70303

**Published:** 2026-06-19

**Authors:** Md. Nafiul Islam, Sumaiya Abedin, Ahbab Mohammad Fazle Rabbi, Md Ismail Tareque

**Affiliations:** ^1^ Department of Population Science and Human Resource Development University of Rajshahi Rajshahi Bangladesh; ^2^ Department of Population Sciences University of Dhaka Dhaka Bangladesh; ^3^ Department of Geography, College of Arts & Social Sciences Sultan Qaboos University Muscat Sultanate of Oman

**Keywords:** Bangladesh, child mortality, Demographic and Health Survey, infant mortality

## Abstract

Despite substantial national progress in reducing child mortality, disparities persist across sociodemographic groups in Bangladesh. A comprehensive understanding of how mortality rates vary by key risk factors is essential to guide equitable health interventions. This study analyzes trends in neonatal, infant, and under‐5 mortality rates in Bangladesh from 1993 to 2022, focusing on the influence of socioeconomic, demographic, and maternal factors. Using data from nine rounds of the Bangladesh Demographic and Health Survey (1993–2022), we estimated mortality rates across categories of factors, such as maternal age, education, household wealth, fertility practices, and region. The findings show a substantial decline in all three mortality indicators over time, although some disparities remain. The neonatal mortality rate decreased across all groups, with the sharpest reduction among mothers under 20 years (from 67 to 23 per 1000 live births) and those with one child (from 44 to 10). Infant mortality rate (IMR) also declined significantly, particularly among wealthier, more educated, and rural mothers. Despite overall improvement, regional disparities persisted, with Khulna reporting the lowest IMR (40 in 2022). Under‐5 mortality rate saw the largest decline among mothers having their first birth before age 20 (from 144 to 34 per 1000) and families with one child (from 98 to 17). Persistent relative risks of around twofold among the poorest households and children of less educated mothers indicate that mortality reductions have disproportionately benefited advantaged groups. These patterns call for prioritizing maternal education, poverty‐targeted service delivery, and geographically focused interventions in high‐risk regions, such as Sylhet and Rangpur, where excess risks remain consistently elevated.

## Graphical abstract

Considerable national reductions in child mortality have been accompanied by persistent disparities across sociodemographic groups in Bangladesh. Using DHS data, this study analyzes trends in neonatal, infant, and under‐5 mortality rates in Bangladesh from 1993 to 2022, focusing on the influence of socioeconomic, demographic, and maternal factors. Results show substantial declines in all mortality indicators over time, but persistent disparities by wealth, education, fertility behavior, and region.

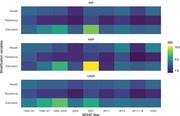

## 1 Introduction

Child mortality rates, particularly the neonatal mortality rate (NMR), infant mortality rate (IMR), and under‐5 mortality rate (U5MR), are crucial public health indicators that play a significant role in evaluating a nation's overall health [1]. The NMR represents the probability of a newborn dying within the first month of life, the IMR represents the probability of dying between birth and the first birthday, and the U5MR represents the probability of dying between birth and the fifth birthday. Recognizing the importance of reducing child mortality, Sustainable Development Goals (SDGs) Target 3.2 aims to decrease the NMR to at least 12 per 1000 live births and the U5MR to at least 25 per 1000 live births by 2030 [2]. Although considerable global progress has been made in reducing both the NMR and U5MR, this progress has not been consistent across all countries. From 1990 to 2019, the average reductions in NMR and U5MR were 52% and 59%, respectively [3]. In line with global trends, Bangladesh has significantly reduced its NMR, IMR, and U5MR over the past three decades. From 1993–94 to 2022, the NMR, IMR, and U5MR fell by 58%, 69%, and 75%, respectively [4]. On the basis of trends in U5MR from 1993 to 2014, a study suggests that Bangladesh is on track to achieve SDG Target 3.2 ahead of the 2030 deadline [5]. However, it is important to note that the decline in these rates was notably slow or stagnant between 2011 and 2017–18 [4].

Previous studies found several risk factors associated with child mortality in different settings [6, 7, 8, 9]. For instance, findings from the World Fertility Survey revealed that a mother's age at childbirth, level of educational attainment, and household income were associated with child mortality in 39 countries, including Bangladesh [10, 11]. Mothers who experience a teen pregnancy were reported to have a higher prevalence of infant and/or child mortality [11]. Moreover, mothers with lower levels of education exhibit a heightened risk of child mortality in comparison to their more educated counterparts [12]. In the least developed countries, including Bangladesh, high rates of NMR, IMR, and U5MR can be considerably mitigated by improving maternal healthcare services, addressing educational deficiencies, and tackling issues related to adolescent fertility [8, 13, 14]. Research also indicates that disparities in healthcare access between urban and rural populations and variations across administrative divisions within Bangladesh significantly contribute to child mortality rates [15, 16, 17].

Although previous studies have provided insight into general trends and factors influencing child mortality, a substantial gap remains in understanding mortality rates when analyzed by specific risk factors. For example, although it is acknowledged that mothers under the age of 20 face increased risks, comprehensive data comparing NMR, IMR, and U5MR among this demographic versus mothers aged 20 and above is insufficient. Additionally, the existing data on these mortality rates in Bangladesh, categorized by socioeconomic and demographic variables, is inadequate. This gap underscores the urgent need for an in‐depth analysis of child mortality rates based on various risk factors to identify vulnerable populations and enhance interventions aimed at reducing child mortality in alignment with SDG Target 3.2 [2]. This study aims to address this gap by analyzing Bangladesh's NMR, IMR, and U5MR from 1993 to 2022 and estimating them by the categories of relevant sociodemographic factors. The objective is to identify at‐risk groups and generate meaningful insights to inform targeted interventions to lower child mortality rates effectively.

## 2 Data and Methods

### 2.1 Data

The data for this study were obtained from the Demographic and Health Surveys (DHS) Program database. The DHS Program has conducted nationally representative surveys on health and family planning in more than 90 low‐ and middle‐income countries. These surveys are cross‐sectional in nature. For this study, we utilized all nine available rounds of the standard Bangladesh DHS (BDHS). Bangladesh's National Institute for Population Research and Training (NIPORT) of the Ministry of Health and Welfare authorizes these surveys, which are funded by USAID [4]. Data collection utilized a two‐stage stratified cluster sampling method and included multiple questionnaires (six for the 2022 BDHS), targeting households, women of reproductive age (15–49), and men aged 18 and older, including detailed birth histories of the mothers [4]. The nine rounds of BDHS we considered for the current study, started with the first round in 1993–94, followed by the second round in 1996–97, the third round in 1999–2000, the fourth round in 2004, the fifth round in 2007, the sixth round in 2011, the seventh round in 2014, the eighth round in 2017–18, and the ninth round—the latest survey—in 2022. In total, 137,657 women were interviewed across these nine surveys, with the following sample sizes: 1993–94 (*n* = 9640), 1996–97 (*n* = 9127), 1999–2000 (*n* = 10,544), 2004 (*n* = 11,440), 2007 (*n* = 10,996), 2011 (*n* = 17,842), 2014 (*n* = 17,863), 2017–18 (*n* = 20,127), and 2022 (*n* = 30,078) [4, 18, 19, 20, 21, 22, 23, 24, 25].

In the DHS datasets, each woman aged 15–49 years who participates in the survey provides a detailed birth history. This includes the month and year of each live birth, the child's survival status, and, when applicable, the age at death, recorded in days, months, or years. To adhere to DHS analytical guidelines and reduce recall bias, our analysis focused on births that occurred within the 5 years (≤59 months) preceding the survey date. The process was implemented as follows: (i) The DHS datasets consist of two date variables: the date of the interview (in century month code, CMC) and the date of the child's birth (also in CMC); (ii) we calculated the interval in months between these two dates for each child documented in the birth history file; and (iii) births with an interval of ≤59 months were included for mortality estimation, whereas those exceeding this interval were excluded. By applying this restriction consistently across all nine rounds of the BDHS (1993–94 to 2022), we ensured comparability across survey years and minimized the risk of errors arising from retrospective misreporting.

### 2.2 Methods

#### 2.2.1 Variables and Measures

##### 2.2.1.1 Outcome Variables (NMR, IMR, and U5MR) and Their Estimation

We considered NMR, IMR, and U5MR as the outcome variables in the current study. Following standardized methodologies established by the DHS, NMR, IMR, and U5MR are estimated [26]. The formulas of NMR, IMR, and U5MR are as follows:

(1) $$\mathrm{NMR}=\frac{D_{0\;\mathrm{month}}}{B}\times 1000,$$

(2) $$\mathrm{IMR}=\frac{D_{1}}{B}\times 1000,$$

(3) $$U5\mathrm{MR}=\frac{D_{5}}{B}\times 1000,$$

where $D_{0\;\mathrm{month}}$, $D_{1},$ and $D_{5}$ are the number of deaths before 1 month of age, under 1 year of age, and under 5 years of age for a specific year, respectively; and B denotes the number of live births in the same year. For each of these measures, the denominators refer to the number of surviving children at the beginning of the specified age range during the specified time period [26]. Estimates of these measures with 95% confidence intervals (CIs) by the categories of sociodemographic variables (defined below) were obtained using the Stata module SYNCMRATES [27].

The SYNCMRATES module employs the direct estimation method introduced by Rutstein and Rojas [26], utilizing a synthetic cohort life table approach. To ensure statistical validity following the DHS's stratified cluster sampling design, we implemented the Taylor series linearization method, which is incorporated within the complex survey design specifications of the DHS datasets (using SVY settings in Stata). The calculation involves several components: (i) sampling weights to adjust for unequal selection probabilities and non‐response, (ii) primary sampling units (clusters) to account for intra‐cluster correlation, and (iii) stratification variables to represent the survey's design strata accurately. This methodology yields point estimates of mortality rates along with design‐adjusted standard errors, from which 95% CIs are derived. By effectively leveraging the complex survey variables provided by DHS, these estimates accurately reflect both sampling and design effects, significantly enhancing their robustness for inferential analysis.

##### 2.2.1.2 Sociodemographic Variables

The analysis examines differentials in NMR, IMR, and U5MR across a range of sociodemographic variables. These variables are used to stratify the population and assess variations in mortality across groups over time, rather than to estimate causal effects. Factors associated with NMR, IMR, and U5MR were selected on the basis of extant literature [6, 7, 8, 9]. The following sociodemographic variables were considered: the mother's age at birth (<20, 20–34, and >34 years), total number of children ever born at the time of the survey (1, 2–3, and >3 live births), preceding birth interval (for non‐first births, categorized as <24 months and ≥24 months), education (no education, primary, secondary, and higher education), employment status (employed and unemployed), wealth index (poor, middle, and rich), place of residence (rural and urban), religion (non‐Muslim and Muslim), exposure to mass media (not exposed and exposed), and administrative division (Barisal, Chattogram, Dhaka, Mymensingh, Khulna, Rajshahi, Rangpur, and Sylhet).

Mothers are classified as “employed” if they participate in any income‐generating activities, whereas those who do not are categorized as “unemployed.” The wealth index is calculated using the standard classification defined in the BDHS [28], wherein the bottom 40% of the wealth quintile (the “poorest” and “poorer” groups) are labeled as “poor,” the subsequent 40% (the “middle” and “richer” groups) are categorized as “middle,” and the remaining 20% (the “richest” group) are classified as “rich.” Mothers who reported reading newspapers, listening to the radio, or watching television at least once a week are considered to have media exposure, whereas those who did not engage in any of these activities at that frequency are considered not exposed to media. Administrative divisions were included to capture broad geographic variation in mortality patterns. These divisions represent standard policy and administrative units used in BDHS reporting. Although divisions are internally heterogeneous, they provide a useful framework for examining regional disparities and facilitating temporal comparisons.

#### 2.2.2 Relative Risk (RR) for Between‐Group Comparisons

To assess relative differences in mortality between groups, RR is estimated for NMR, IMR, and U5MR. The RR is defined as the ratio of the mortality rates in a disadvantaged group to those in an advantaged (reference) group. The formula used to calculate RR ratio is as follows:

$$\mathrm{RR}=\frac{M_{d}}{M_{a}},$$

where $M_{d}$ denotes the mortality rate in the disadvantaged group, and $M_{a}$ represents the corresponding rate in the advantaged group.

The RR ratios are used to examine differences between groups over time across key dimensions (wealth, education, and residence) [29]. For wealth‐related comparisons, the poor and rich groups of our wealth index variable were used to represent disadvantaged and advantaged groups, respectively. Educational differences were examined by comparing mothers with no formal education to those with higher education, whereas variations by place of residence were assessed by contrasting rural and urban populations.

We also calculated 95% confidence intervals (CI) for RR using the following formula, which yields asymmetric intervals appropriate for risk estimates. The formula used is as follows:

$$95\%\;\mathrm{CI}\;\mathrm{for}\;\mathrm{RR}=\exp\left[\ln\left(\mathrm{RR}\right)\pm 1.96\times \mathrm{SE}\left\{\ln\left(\mathrm{RR}\right)\right\}\right].$$

To estimate the CI for RR, we first calculated the natural logarithm of the RR (i.e., ln(RR)) and then the standard error of ln(RR) using the delta method for RRs derived from proportions. We then calculated the CI on the log scale and back‐transformed the results by exponentiation.

### 2.3 Ethical Statement

This study involved the participation of human subjects; however, the authors of this manuscript were not directly engaged in the data collection processes. The survey was conducted by NIPORT, Mitra and Associates, and ICF International, with trainee staffs executing the interviews. Verbal consent was obtained from each respondent prior to the interviews, in accordance with an informed consent statement. The ICF Institutional Review Board provided ethical approval for the BDHS data, adhering to the ethical standards established by the Helsinki Declaration (1964). For this study, we registered online and agreed to all terms and conditions regarding the use of the BDHS datasets.

## 3 Results

### 3.1 Trends in NMR, IMR, and U5MR

**FIGURE 1:**
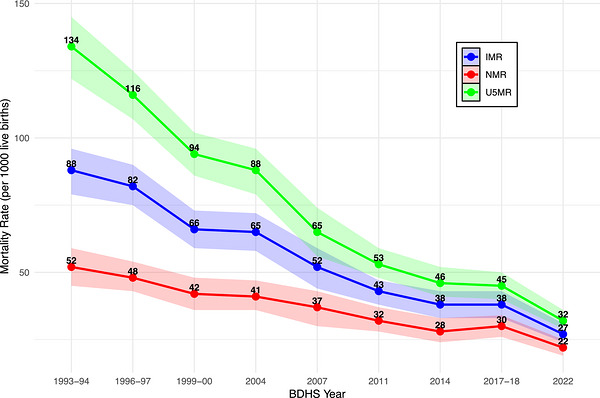
Trends in infant mortality rate (IMR), neonatal mortality rate (NMR), and under‐5 mortality rate (U5MR) per 1000 live births in Bangladesh from 1993 to 2022 (BDHS). The shaded areas around the trend lines represent the 95% CIs for each rate.

The trends in NMR, IMR, and U5MR in Bangladesh show a significant decline over time, except for a temporary rise in NMR during 2017–18 (Figure 1). NMR declined steadily from 52 to 22 deaths per 1000 live births from 1993–94 to 2022, whereas IMR and U5MR fell from 88 and 134 to 27 and 32, respectively, during the same period.

**FIGURE 2:**
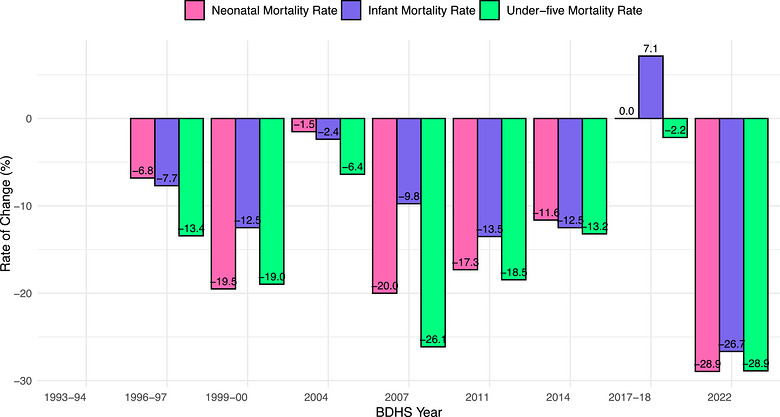
Rate of change over time in infant mortality rate (IMR), neonatal mortality rate (NMR), and under‐5 mortality rate (U5MR) per 1000 live births in Bangladesh from 1993 to 2022 (BDHS). A negative rate indicates a decrease from the previous survey, whereas a positive rate indicates an increase.

The rates of decline for NMR, IMR, and U5MR during this period were not uniform (Figure 2). From 1993–94 to 2022, NMR declined by 57.7%, IMR by 69.3%, and U5MR by 76.1% in Bangladesh. Initially, the pace of decline was slower for all three measures. The most notable reduction occurred between 2017–18 and 2022, during which NMR fell by 26.7%, whereas both IMR and U5MR decreased by 28.9%.

### 3.2 Trends in NMR by Categories of Sociodemographic Variables

The NMR by the categories of sociodemographic variables from 1993–94 to 2022 is shown in Table 1. Overall, NMR has declined significantly across categories, with the highest rates among mothers aged under 20, dropping from 67 per 1000 live births in 1993–94 to 23 per 1000 live births in 2022. Conversely, NMR is lowest among women who have one child, decreasing from 44 in 1993–94 to 10 in 2022, whereas those with three or more children exhibit greater variability across the years. Shorter birth intervals, especially under 24 months, are associated with higher NMR, peaking at 75 in 1999–2000; however, intervals of 24 months or more show a decline from 37 in 1993–94 to 21 in 2022. Higher education is associated with greater NMR reductions from 42 in 1993–94 to 14 in 2022. Meanwhile, women without formal education continue to experience relatively high NMR, though it has also decreased over time, albeit at a slower pace. The NMR among unemployed mothers is marginally higher than for employed women, but both groups have experienced a decline over time. The NMR decreases across wealth categories, with rich individuals demonstrating the lowest rates over time. Generally, rural areas exhibit higher NMR than urban areas, though both have declined over time. Additionally, the NMR among Muslims remains consistently higher than that of non‐Muslims, yet both groups have shown a decrease over the years. Women exposed to mass media have lower NMR, which has declined over time. Significant regional disparities persist, with historically high rates in Barisal and Sylhet, which have recently decreased.

**TABLE 1:** Neonatal mortality rate (per 1000 live births) by categories of sociodemographic variables in Bangladesh from 1993 to 2022.

Variables	NMR (95% CI)								
	BDHS 1993–94	BDHS 1996–97	BDHS 1999–2000	BDHS 2004	BDHS 2007	BDHS 2011	BDHS 2014	BDHS 2017–18	BDHS 2022
**Mother's age at birth**									
<20 years	67 (54–79)	58 (48–69)	60 (49–71)	53 (42–63)	51 (40–63)	45 (36–53)	31 (23–39)	40 (31–48)	23 (18–28)
20–34 years	43 (36–50)	42 (36–49)	34 (28–40)	34 (28–41)	29 (22–36)	25 (20–30)	27 (21–34)	26 (21–31)	22 (18–25)
>34 years	73 (46–100)	52 (22–83)	12 (2–22)	43 (18–67)	17 (0–34)	28 (9–47)	17 (2–32)	22 (3–42)	30 (10–49)
**Children ever born**									
1	44 (32–56)	29 (19–39)	43 (30–55)	31 (21–41)	21 (11–31)	16 (10–22)	16 (10–21)	16 (11–22)	10 (7–13)
2–3	56 (46–66)	50 (41–59)	47 (39–55)	42 (33–50)	38 (30–47)	41 (34–48)	34 (27–41)	36 (30–42)	26 (22–30)
>3	52 (43–61)	56 (45–67)	34 (26–43)	48 (36–59)	48 (32–64)	32 (23–42)	34 (23–44)	40 (26–54)	38 (25–50)
**Preceding birth interval**									
<24 months	78 (61–96)	66 (48–83)	54 (35–74)	53 (28–79)	79 (50–108)	45 (28–61)	22 (7–37)	45 (28–63)	26 (13–38)
≥24 months	37 (31–43)	39 (32–46)	23 (18–28)	29 (23–35)	22 (16–28)	23 (18–28)	26 (19–33)	22 (17–27)	21 (17–25)
**Level of education**									
No education	57 (48–67)	50 (42–58)	46 (38–54)	47 (37–57)	44 (28–60)	32 (22–41)	26 (16–36)	35 (19–51)	28 (16–40)
Primary	47 (37–58)	50 (38–61)	37 (27–46)	40 (30–49)	37 (27–48)	36 (29–43)	31 (21–40)	35 (26–44)	23 (16–29)
Secondary	40 (26–53)	42 (27–57)	46 (33–58)	36 (25–48)	36 (25–46)	31 (24–37)	31 (23–38)	29 (24–35)	24 (20–28)
Higher	42 (8–77)	23 (−10 to 56)	11 (−2 to 24)	32 (11–52)	4 (−2 to 11)	29 (8–49)	13 (4–21)	21 (12–31)	14 (8–19)
**Employment status**									
Unemployed	51 (44–58)	52 (44–59)	41 (35–47)	41 (35–47)	38 (31–46)	31 (26–35)	27 (22–33)	28 (23–33)	21 (17–25)
Employed	59 (42–76)	42 (33–51)	44 (32–57)	44 (31–57)	32 (21–43)	49 (33–65)	31 (23–39)	33 (26–40)	25 (17–32)
**Wealth index**									
Poor	64 (54–74)	48 (40–56)	46 (38–55)	41 (32–49)	40 (29–52)	36 (29–43)	35 (27–42)	31 (24–37)	30 (25–36)
Middle	46 (36–55)	58 (48–68)	41 (33–49)	43 (34–53)	37 (27–47)	33 (26–40)	29 (20–38)	31 (24–38)	18 (15–22)
Rich	34 (24–43)	25 (15–35)	30 (19–41)	38 (27–50)	26 (16–37)	23 (14–31)	14 (8–20)	27 (18–37)	12 (7–17)
**Place of residence**									
Urban	32 (21–43)	42 (27–56)	39 (27–50)	39 (29–49)	29 (20–38)	32 (23–40)	21 (14–28)	35 (27–44)	22 (17–27)
Rural	55 (47–62)	49 (43–55)	43 (37–49)	42 (36–48)	39 (31–47)	33 (28–38)	31 (25–36)	28 (24–33)	22 (19–26)
**Religion**									
Muslim	53 (46–59)	48 (42–54)	41 (36–47)	42 (36–48)	38 (31–45)	33 (28–37)	29 (25–34)	29 (25–33)	22 (19–25)
Non‐Muslim	49 (29–69)	51 (31–71)	48 (30–65)	37 (20–53)	22 (9–35)	28 (15–41)	17 (1–34)	42 (26–58)	20 (11–29)
**Exposure to media**									
No exposure	60 (50–69)	48 (40–55)	44 (37–51)	44 (35–52)	36 (27–46)	33 (27–39)	35 (27–42)	29 (23–35)	24 (20–28)
Exposed	42 (34–50)	49 (41–58)	39 (32–47)	40 (32–47)	37 (29–45)	32 (26–38)	22 (15–29)	31 (26–37)	20 (16–24)
**Division^a^**									
Barisal	55 (36–74)	47 (30–63)	37 (19–54)	28 (16–39)	33 (21–46)	38 (24–51)	21 (9–33)	32 (20–44)	18 (12–24)
Chattogram	51 (39–64)	42 (31–52)	31 (21–41)	35 (25–46)	35 (20–51)	21 (14–28)	24 (16–32)	31 (23–39)	23 (15–31)
Dhaka	50 (38–61)	44 (33–55)	45 (34–56)	44 (33–56)	32 (21–43)	36 (27–45)	25 (16–34)	33 (23–42)	19 (13–25)
Khulna	55 (35–74)	53 (34–72)	36 (26–47)	36 (22–50)	30 (17–44)	32 (18–45)	41 (24–58)	24 (13–34)	18 (11–24)
Mymensingh	—	—	—	—	—	—	—	24 (15–32)	25 (18–33)
Rajshahi	55 (39–72)	52 (40–64)	43 (31–54)	36 (22–50)	43 (27–58)	39 (27–50)	31 (18–44)	26 (14–38)	20 (12–28)
Rangpur	—	—	—	—	—	27 (16–38)	27 (14–40)	37 (22–52)	30 (21–40)
Sylhet	—	76 (58–95)	72 (55–90)	58 (42–74)	50 (31–69)	45 (31–59)	39 (29–50)	31 (18–43)	29 (19–39)

Abbreviations: BDHS, Bangladesh Demographic and Health Surveys; CI, confidence interval; NMR, neonatal mortality rate.

^a^Divisions with data unavailable were recently created. For an instance, Mymensingh Division was part of Dhaka Division until September 2015.

### 3.3 Trends in IMR by Categories of Sociodemographic Variables

Table 2 displays IMR by the categories of sociodemographic variables from 1993 to 2022. With a few exceptions, the IMR for each category of sociodemographic variables declined significantly during this period. The rates experienced fluctuations, peaking at 121 in 1993–94, before decreasing to 40 by 2022 for mothers who had their first births in their late 30 s. Higher maternal education was associated with larger declines; the IMR for mothers with secondary education decreased from 51 to 29, while for those with higher education, it fell from 51 to 18 between 1993–94 and 2022. The most pronounced reduction in IMR was observed among the rich mothers, whereas the decline was slower among middle‐income and poor groups. Rural mothers reported a quicker decline in IMR compared to their urban counterparts. Additionally, there were regional disparities in IMR reductions, with certain divisions experiencing more rapid declines than others. The lowest IMR recorded in 2022 was in Khulna.

**TABLE 2:** Infant mortality rate (per 1000 live births) by categories of sociodemographic variables in Bangladesh from 1993 to 2022.

Variables	IMR (95% CI)								
	BDHS 1993–94	BDHS 1996–97	BDHS 1999–2000	BDHS 2004	BDHS 2007	BDHS 2011	BDHS 2014	BDHS 2017–18	BDHS 2022
**Mother's age at birth**									
<20 years	103 (89–117)	94 (81–106)	84 (71–96)	72 (60–83)	67 (53–81)	56 (46–65)	41 (32–50)	47 (38–56)	29 (23–35)
20–34 years	76 (68–85)	75 (66–83)	58 (50–66)	60 (51–68)	43 (34–52)	35 (29–40)	36 (29–43)	34 (28–40)	26 (22–30)
>34 years	121 (89–154)	97 (59–134)	41 (20–62)	82 (53–112)	38 (16–60)	47 (22–71)	40 (9–71)	22 (3–42)	40 (18–61)
**Children ever born**									
1	70 (55–86)	50 (37–62)	52 (39–65)	44 (31–57)	28 (17–39)	19 (13–26)	19 (13–25)	17 (12–22)	14 (10–17)
2–3	87 (75–99)	81 (71–92)	72 (62–81)	61 (52–71)	52 (42–62)	51 (43–58)	46 (39–54)	43 (36–50)	31 (26–35)
>3	94 (82–106)	98 (85–111)	67 (55–78)	83 (69–97)	72 (54–90)	52 (40–64)	48 (34–61)	63 (47–79)	48 (35–61)
**Preceding birth interval**									
<24 months	140 (121–160)	128 (106–151)	87 (64–109)	86 (54–118)	118 (84–152)	66 (47–86)	30 (13–47)	56 (36–75)	39 (23–56)
≥24 months	66 (57–74)	66 (58–74)	45 (38–52)	54 (46–62)	34 (27–42)	33 (27–39)	36 (28–44)	31 (25–37)	26 (21–30)
**Level of education**									
No education	99 (89–110)	91 (81–101)	75 (64–86)	77 (65–90)	70 (51–88)	55 (43–66)	38 (26–50)	43 (26–61)	34 (21–47)
Primary	79 (67–91)	79 (65–93)	63 (51–75)	61 (49–73)	52 (40–63)	45 (38–53)	43 (32–53)	47 (37–57)	30 (23–37)
Secondary	51 (36–66)	58 (40–76)	59 (45–72)	55 (41–69)	43 (32–55)	36 (30–43)	40 (32–48)	36 (30–42)	29 (24–33)
Higher	51 (13–89)	34 (−4 to 73)	21 (3–39)	45 (22–68)	11 (0–23)	30 (9–50)	17 (7–27)	24 (14–33)	18 (11–25)
**Employment status**									
Unemployed	86 (77–95)	87 (78–96)	65 (58–73)	64 (56–71)	54 (44–64)	40 (35–45)	36 (31–42)	37 (31–42)	26 (22–31)
Employed	93 (72–114)	73 (61–85)	70 (55–85)	72 (55–90)	43 (32–55)	67 (49–86)	43 (32–53)	40 (32–47)	33 (24–41)
**Wealth index**									
Poor	106 (94–118)	87 (77–98)	76 (65–86)	67 (55–78)	59 (45–72)	50 (43–58)	47 (38–55)	40 (34–47)	36 (30–41)
Middle	77 (66–88)	87 (77–98)	61 (51–71)	65 (54–76)	51 (40–62)	40 (32–47)	35 (26–45)	38 (30–46)	23 (19–28)
Rich	57 (43–70)	52 (38–66)	49 (36–62)	62 (48–76)	35 (23–46)	29 (20–38)	24 (16–33)	32 (22–42)	18 (12–24)
**Place of residence**									
Urban	59 (43–76)	73 (54–92)	63 (48–78)	69 (56–83)	40 (30–51)	42 (33–51)	34 (26–42)	42 (33–51)	27 (21–34)
Rural	90 (82–99)	83 (76–91)	67 (60–74)	64 (56–72)	54 (45–64)	43 (37–48)	40 (34–45)	36 (31–42)	27 (24–31)
**Religion**									
Muslim	88 (80–96)	82 (75–89)	65 (58–72)	66 (59–73)	53 (45–61)	43 (38–48)	38 (33–43)	37 (32–42)	28 (24–31)
Non‐Muslim	80 (57–103)	87 (61–114)	75 (55–95)	54 (30–78)	34 (21–48)	38 (23–52)	37 (19–56)	48 (32–63)	24 (13–35)
**Exposure to media**									
No exposure	98 (87–109)	88 (79–97)	73 (63–82)	71 (59–82)	54 (42–66)	45 (39–52)	45 (36–53)	39 (32–46)	29 (24–34)
Exposed	72 (63–82)	75 (65–85)	57 (48–67)	61 (52–70)	50 (40–59)	39 (33–46)	32 (24–39)	37 (31–43)	26 (21–30)
**Division^a^**									
Barisal	94 (70–119)	90 (66–114)	64 (39–89)	54 (36–72)	46 (31–61)	49 (33–65)	26 (14–38)	41 (29–54)	24 (16–32)
Chattogram	90 (75–105)	74 (60–87)	50 (37–62)	57 (45–70)	50 (35–65)	35 (27–42)	36 (28–45)	33 (24–41)	26 (18–34)
Dhaka	92 (77–106)	80 (66–93)	75 (62–87)	72 (57–87)	47 (31–62)	44 (33–54)	35 (25–46)	41 (29–52)	25 (17–33)
Khulna	81 (61–100)	75 (54–96)	47 (33–60)	48 (33–63)	43 (27–60)	36 (23–50)	47 (31–64)	32 (21–43)	19 (12–26)
Mymensingh	—	—	—	—	—	—	—	32 (23–42)	30 (23–38)
Rajshahi	80 (62–97)	83 (68–98)	66 (52–80)	48 (33–63)	53 (36–70)	51 (38–65)	38 (24–51)	37 (21–52)	26 (17–35)
Rangpur	—	—	—	—	—	36 (23–48)	34 (20–49)	40 (25–55)	33 (24–43)
Sylhet	—	124 (99–150)	112 (89–135)	97 (72–122)	80 (56–104)	59 (44–74)	55 (44–66)	52 (39–65)	46 (33–59)

Abbreviations: BDHS, Bangladesh Demographic and Health Surveys; CI, confidence interval; IMR, infant mortality rate.

^a^Divisions with data unavailable were recently created. For an instance, Mymensingh Division was part of Dhaka Division until September 2015.

### 3.4 Trends in U5MR by Categories of Sociodemographic Variables

Table 3 presents the U5MR by categories of sociodemographic variables from 1993–94 to 2022. Remarkably, mothers who had their first child before age 20 experienced the largest decline in U5MR, which decreased from 144 (95% CI: 128–160) in 1993–94 to 34 (95% CI: 27–40) in 2022. The largest reduction in the IMR was observed in families with only one child, where the U5MR plummeted from 98 in 1993–94 to 17 in 2022. In contrast, families with more than three children saw a decrease in U5MR from 150 in 1993–94 to 58 in 2022, although a slight increase was noted after 2011. Significant declines in U5MR were observed across all educational levels. For mothers without formal education, the rate fell from 154 in 1993–94 to 38 in 2022, and those with higher education experienced the most pronounced decline, from 61 to 19. Across wealth categories, U5MR decreased from 95 to 41 among the poor, from 87 to 28 for the middle class, and from 66 to 22 for the rich. Notably, non‐Muslim mothers experienced a more significant reduction, with their U5MR falling from 137 in 1993–94 to 28 in 2022. Interestingly, mass media exposure did not show any discernible impact on U5MR. The region with the most substantial decline in U5MR is Khulna, where the rate decreased significantly from 102 in 1993–94 to 22 in 2022. In contrast, Sylhet experienced the weakest trend, with its U5MR reducing only from 162 to 52 over the same period.

**TABLE 3:** Under‐5 mortality rate (per 1000 live births) by categories of sociodemographic variables in Bangladesh from 1993 to 2022.

Variables	U5MR (95% CI)								
	BDHS 1993–94	BDHS 1996–97	BDHS 1999–2000	BDHS 2004	BDHS 2007	BDHS 2011	BDHS 2014	BDHS 2017–18	BDHS 2022
**Mother's age at birth**									
<20 years	144 (128–160)	126 (112–141)	106 (93–119)	91 (77–105)	82 (68–97)	67 (56–77)	49 (40–58)	54 (45–63)	34 (27–40)
20–34 years	126 (114–137)	108 (98–119)	90 (80–99)	83 (72–93)	55 (46–65)	45 (38–52)	45 (37–52)	41 (35–47)	31 (27–36)
>34 years	161 (126–197)	131 (90–173)	65 (39–91)	117 (82–152)	51 (20–82)	62 (34–90)	46 (14–77)	23 (4–42)	43 (21–65)
**Children ever born**									
1	98 (75–121)	65 (49–81)	61 (46–76)	54 (37–70)	35 (23–48)	21 (14–28)	22 (16–29)	21 (15–28)	17 (12–21)
2–3	122 (108–137)	108 (95–120)	87 (77–98)	76 (65–86)	63 (52–74)	61 (53–68)	54 (46–61)	48 (41–55)	35 (30–40)
>3	150 (135–164)	139 (123–154)	110 (96–124)	117 (100–134)	91 (72–110)	70 (57–83)	61 (47–76)	78 (60–96)	58 (44–72)
**Preceding birth interval**									
<24 months	206 (183–228)	171 (146–196)	129 (104–154)	125 (92–158)	146 (109–183)	82 (61–103)	46 (25–66)	77 (53–101)	48 (31–65)
≥24 months	110 (99–121)	99 (88–110)	74 (65–84)	75 (66–84)	45 (37–54)	45 (38–51)	43 (35–52)	37 (30–43)	31 (26–36)
**Level of education**									
No education	154 (140–168)	131 (119–143)	110 (98–122)	108 (93–123)	86 (65–106)	71 (58–84)	50 (36–64)	55 (36–74)	38 (25–51)
Primary	109 (94–125)	106 (91–121)	87 (74–100)	78 (65–92)	64 (51–77)	55 (46–63)	50 (39–60)	53 (43–64)	35 (27–42)
Secondary	88 (67–110)	76 (57–95)	74 (59–88)	69 (54–84)	55 (42–68)	46 (38–53)	47 (38–56)	43 (36–50)	35 (30–40)
Higher	61 (21–101)	34 (−4 to 73)	21 (3–39)	49 (25–74)	22 (8–37)	36 (15–57)	22 (11–34)	27 (17–38)	19 (12–27)
**Employment status**									
Unemployed	131 (119–142)	122 (110–133)	91 (83–99)	84 (75–93)	68 (58–79)	51 (45–57)	44 (38–50)	45 (39–52)	31 (26–35)
Employed	149 (124–173)	105 (90–119)	105 (87–122)	102 (83–120)	55 (42–69)	75 (55–95)	51 (40–63)	45 (37–53)	38 (29–47)
**Wealth index**									
Poor	166 (150–182)	127 (113–140)	112 (100–124)	95 (82–109)	71 (56–85)	64 (56–73)	57 (48–67)	49 (42–57)	41 (35–47)
Middle	115 (101–129)	121 (108–134)	83 (72–94)	87 (74–99)	69 (57–82)	48 (40–57)	42 (32–52)	44 (36–53)	28 (23–32)
Rich	79 (64–94)	69 (50–88)	63 (50–76)	66 (52–81)	42 (29–55)	37 (26–48)	30 (21–39)	36 (26–47)	22 (16–29)
**Place of residence**									
Urban	91 (72–110)	96 (70–122)	82 (66–97)	92 (76–108)	50 (39–61)	50 (40–59)	37 (29–46)	48 (39–58)	31 (25–37)
Rural	138 (126–150)	118 (108–127)	97 (88–105)	86 (77–96)	69 (58–80)	55 (48–61)	49 (43–56)	43 (38–49)	33 (29–37)
**Religion**									
Muslim	133 (122–144)	116 (107–125)	94 (85–102)	90 (81–98)	66 (57–76)	54 (49–60)	47 (41–52)	44 (39–50)	33 (29–36)
Non‐Muslim	137 (105–169)	115 (85–146)	98 (75–120)	61 (36–87)	49 (29–68)	42 (28–57)	41 (22–60)	48 (32–65)	28 (17–40)
**Exposure to media**									
No exposure	148 (134–163)	129 (117–140)	109 (98–120)	101 (88–114)	69 (56–82)	59 (51–66)	55 (46–65)	48 (41–56)	35 (29–40)
Exposed	112 (99–125)	100 (87–112)	73 (63–83)	76 (66–86)	61 (51–72)	48 (40–55)	37 (30–45)	42 (35–49)	30 (25–35)
**Division^a^**									
Barisal	137 (111–164)	128 (102–154)	96 (66–126)	82 (55–108)	65 (45–84)	62 (45–80)	35 (21–49)	50 (37–64)	37 (25–49)
Chattogram	156 (134–178)	116 (98–134)	85 (68–102)	89 (72–107)	72 (54–90)	50 (40–60)	50 (38–62)	41 (31–52)	32 (24–41)
Dhaka	137 (120–154)	116 (97–135)	102 (87–116)	97 (80–115)	57 (39–75)	54 (43–65)	41 (30–52)	48 (36–60)	27 (19–34)
Khulna	102 (76–127)	83 (61–106)	60 (45–74)	58 (42–75)	55 (38–72)	40 (26–55)	56 (39–73)	36 (24–47)	22 (15–30)
Mymensingh	—	—	—	—	—	—	—	37 (27–47)	34 (26–42)
Rajshahi	112 (90–134)	111 (94–129)	90 (75–105)	58 (42–75)	60 (42–77)	63 (50–77)	43 (28–57)	45 (28–62)	30 (21–39)
Rangpur	—	—	—	—	—	42 (29–55)	39 (23–54)	42 (27–57)	41 (30–52)
Sylhet	—	162 (134–191)	148 (124–173)	117 (88–146)	100 (72–129)	71 (54–88)	67 (55–79)	60 (46–75)	52 (40–64)

Abbreviations: BDHS, Bangladesh Demographic and Health Surveys; CI, confidence interval; U5MR, under‐5 mortality rate.

^a^Divisions with data unavailable were recently created. For an instance, Mymensingh Division was of part Dhaka Division until September 2015.

### 3.5 Percentage Decline in NMR, IMR, and U5MR During 1993–2022

Table 4 summarizes reductions in NMR, IMR, and U5MR by the categories of sociodemographic variables. It is noteworthy that all three indicators demonstrate the largest decreases in smaller families, particularly among mothers with one child. Conversely, the smallest reductions in both NMR and U5MR are found in larger families, where mothers have three or more children. Furthermore, the slowest decline in IMR is observed among mothers with a secondary education.

**TABLE 4:** Percentage decline in NMR, IMR, and U5MR during 1993–2022.

Variables	Decline (%) during 1993–2022		
	NMR	IMR	U5MR
**Mother's age at birth**			
<20 years	66	72	76
20–34 years	49	66	75
>34 years	59	67	73
**Children ever born**			
1	77	80	83
2–3	54	64	71
>3	27	49	61
**Preceding birth interval**			
<24 months	67	72	77
≥24 months	43	61	72
**Level of education**			
No education	51	66	75
Primary	51	62	68
Secondary	40	43	60
Higher	67	65	69
**Employment status**			
Unemployed	59	70	76
Employed	58	65	74
**Wealth index**			
Poor	53	66	75
Middle	61	70	76
Rich	65	68	72
**Place of residence**			
Urban	31	54	66
Rural	60	70	76
**Religion**			
Muslim	58	68	75
Non‐Muslim	59	70	80
**Exposure to media**			
No exposure	60	70	76
Exposed	52	64	73
**Division^a^**			
Barisal	67	74	73
Chattogram	55	71	79
Dhaka	62	73	80
Khulna	67	77	78
Mymensingh	—	—	—
Rajshahi	64	68	73
Rangpur	—	—	—
Sylhet	—	—	—

Abbreviations: IMR, infant mortality rate; NMR, neonatal mortality rate; U5MR, under‐5 mortality rate.

^a^ Divisions with data unavailable were recently created. For an instance, Mymensingh Division was part of Dhaka Division until September 2015.

### 3.6 Trends in Relative Risk of Child Mortality Across Population Groups

**FIGURE 3:**
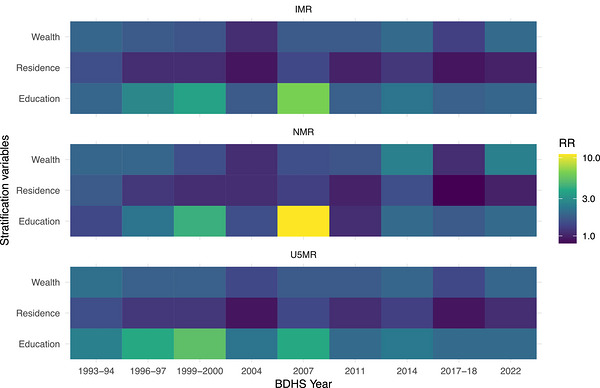
Trends in relative risk of child mortality across education (no education to higher education), wealth index (poor to rich), and place of residence (rural to urban), 1993–2022. IMR, infant mortality rate; NMR, neonatal mortality rate; U5MR, under‐5 mortality rate.

Relative differences in child mortality across education (no education to higher education), wealth index (poor to rich), and place of residence (rural to urban) were assessed using RR over time (Figure 3). Wealth inequality was evident throughout the study period, with children from poor households experiencing around 1.5–2 times higher mortality than those from rich households in the 1990s (e.g., for U5MR, RR: 2.1, 95% CI: 1.6–2.7 in 1993–94 and RR: 1.8, 95% CI: 1.4–2.4 in 1996–97). Although this gap narrowed around 2004 (e.g., for IMR, RR: 1.1, 95% CI: 0.8–1.5), it widened again in later years, with poor households facing nearly double the mortality risk by 2014 and 2022 (e.g., for NMR, RR: 2.5, 95% CI: 1.4–4.6 in 2014 and RR: 2.5, 95% CI: 1.3–4.9 in 2022). Educational inequalities were particularly striking, especially in the late 1990s and again in 2007, as children of uneducated mothers faced substantially higher mortality risks than those whose mothers had higher education. The disparity peaked around 1999–2000 and 2007, with U5MR among children of uneducated mothers being more than five times higher in 1999–2000 (RR: 5.2, 95% CI: 3.3–8.3) and NMR reaching an 11‐fold higher risk in 2007 (RR: 11.0, 95% CI: 4.0–30.5). Although these inequalities declined after 2011, they remained evident in recent surveys, with children of uneducated mothers still experiencing around 1.7–2 times higher mortality risk in 2017–18 and 2022 (e.g., for U5MR, RR: 2.0, 95% CI: 1.3–3.2 in 2017–18 and RR: 2.0, 95% CI: 1.2–3.4 in 2022).

In terms of rural–urban disparities, the differences were comparatively modest throughout the study period. Rural children generally experienced slightly higher mortality risks than urban children during the 1990s (e.g., for NMR, RR: 1.7, 95% CI: 1.1–2.6 in 1993–94 and for U5MR, RR: 1.5, 95% CI: 1.2–1.9). However, these inequalities gradually narrowed over time, with most RRs approaching unity after 2004 and many CIs, including 1.0, indicating limited or nonsignificant differences between rural and urban areas (e.g., for IMR, RR: 0.9, 95% CI: 0.7–1.3 in 2004; for U5MR, RR: 0.9, 95% CI: 0.6–1.3 in 2017–18; and for IMR, RR: 1.0, 95% CI: 0.6–1.7 in 2022).

## 4 Discussion

The observed decline in IMR, NMR, and U5MR over time indicates significant advancements in public health, healthcare accessibility, and socioeconomic conditions. The pace of this decline varies according to factors, such as education, wealth, region, and others, which significantly influence health outcomes for mothers and children. These findings emphasize the complex and multifaceted nature of mortality, where socioeconomic and demographic factors interact to shape overall health outcomes.

The NMR has shown a significant decline across most background characteristics from 1993–94 to 2022, with a few notable exceptions. The trend reflects a gradual reduction in neonatal mortality over time, particularly in areas such as maternal education, birth intervals, and media exposure. Many studies have underscored the advantages of educating mothers in enhancing maternal and child healthcare [10, 11]. Educated mothers are more likely to have access to healthcare and make informed decisions, leading to fewer neonatal deaths [30]. Furthermore, greater intervals between births, providing more time between pregnancies, tend to lower the risk of complications for both mothers and their newborns [31].

Although progress has been made, significant disparities in NMR continue to exist, particularly among rural populations, younger mothers, and those with lower education levels. Notably, younger mothers; especially those under the age of 20; have experienced a steady decline in NMR, decreasing from 67 per 1000 live births in 1993–94 to 23 in 2022. Nevertheless, this age group still has the highest NMR, underscoring the vulnerability of neonatal health among younger mothers. Previous research suggests that this elevated risk may be partially attributed to nutritional deficiencies [32]. Furthermore, another study indicates that adolescent pregnancies, particularly in younger adolescents, can lead to competition for nutrients between the mother and the fetus, as these young mothers may still require additional energy for their own growth [33]. Another important factor influencing neonatal survival is birth spacing. Women with birth intervals of less than 24 months consistently experience higher NMR, with the highest rate recorded at 75 in 1999–2000 before showing a steady decline. In contrast, women with longer birth intervals (24 months or more) have seen a gradual reduction in NMR, falling from 37 in 1993–94 to 21 in 2022. Similar trends have been observed in Bangladesh, where NMR has demonstrated a significant declining pattern as the length of the previous birth interval increases [34].

The trends indicate a substantial reduction in IMR across almost all background characteristics over the past three decades, with notable improvements in areas such as maternal age, education level, wealth, media exposure, and place of residence. A particularly striking finding is the substantial decline in IMR among mothers with higher education, whose rates fell from 51 in 1993–94 to 18 in 2022. Wealthier mothers experienced the steepest decrease in IMR, whereas the reduction for middle‐income and poor mothers was relatively slower. This disparity can be attributed to better access to healthcare services for wealthier mothers, including prenatal care, skilled birth attendants, and postnatal care, which significantly mitigate the risk of infant mortality. Furthermore, wealthier mothers tend to possess higher levels of education, which often translates into improved knowledge regarding maternal health practices, nutrition, and infant care [35]. Interestingly, our findings suggest that rural mothers have reported a quicker decline in IMR compared to their urban counterparts, indicating regional differences in healthcare access and quality. The Government of Bangladesh has long focused on targeted interventions to reduce maternal and child mortality. Over the years, there have been significant improvements in maternal and child health programs aimed at rural populations. Initiatives designed to enhance healthcare access, such as community health workers, mobile clinics, and outreach services, have been effectively implemented in rural areas, leading to a more rapid decline in IMR than seen in urban settings [36]. Regional disparities in IMR have been identified, with certain areas showing more rapid improvements than others. Notably, Khulna achieved the lowest IMR in 2022, underscoring the effectiveness of regional health interventions. These differences indicate that to further enhance infant survival rates nationwide, targeted health strategies tailored to specific regions may be essential.

Although all child mortality indicators are declining in Bangladesh, the pace of decline is much faster for U5MR than NMR and IMR. Interestingly, the sharpest decline in U5MR was observed among mothers who had their first birth before age 20. This finding is different from those in other developing countries, as adolescent pregnancy is generally associated with worse child survival, a factor that is later mitigated by health‐seeking behavior. Although improved access to healthcare and support systems may play a vital role in reducing child mortality, further study is still needed to understand the underlying mechanisms of this phenomenon in Bangladesh. Families with only one child also reported a significant decrease in U5MR, from 98 in 1993–94 to 17 in 2022. In contrast, families with more than three children saw a smaller reduction, with U5MR dropping from 150 to 58, showing a slight increase in mortality after 2011. Families with fewer children are better positioned to allocate more resources—such as time, attention, and finances—to each child. This enables parents to invest more significantly in healthcare services, including regular check‐ups, vaccinations, and prompt treatment for illnesses, all of which are crucial for reducing child mortality [37].

Similar to NMR and IMR, the decline in U5MR is most pronounced among women with higher education, with the rate dropping from 61 in 1993–94 to 19 in 2022. One of the more surprising findings was the lack of impact of mass media exposure on U5MR, as no significant difference was observed between mothers with media exposure and those without. Further study may give us more insight into this. Regional differences were also significant, with Khulna again showing the sharpest decline in U5MR, dropping from 102 in 1993–94 to 22 in 2022. In contrast, Sylhet experienced a slower decline, with U5MR only reducing from 162 to 52 during the same period. Previous study mentioned Sylhet for lagging in several critical associated indicators such as parental education, maternal BMI, and women empowerment, which are significant determinants for U5MR [38]. These findings underscore the necessity for targeted health interventions that address regional disparities and other sociodemographic factors.

### 4.1 Strengths and Limitations

This study analyzed differentials in NMR, IMR, and U5MR in Bangladesh, focusing on sociodemographic factors rather than providing a contemporary analysis of determinants based on the number of deaths. Such an analysis is more robust and unique, as it provides evidence from observed data.

However, this analysis also has some limitations. Being a cross‐sectional study, it does not allow us to identify cohort effects associated with any of these measures. Furthermore, we focused only on selected differentials of these mortality rates; a more comprehensive analysis involving additional background information may yield deeper insights for policy‐making.

## 5 Conclusion

This study documents a significant decline in NMR, IMR, and U5MR in Bangladesh between 1993 and 2022, reflecting sustained improvements in public health and healthcare access. However, these gains have not been equitably shared. Persistent disparities by household wealth, maternal education, and geographic location indicate that reductions in child mortality have predominantly benefited more advantaged groups. Although the study does not establish causal relationships, it provides robust, nationally representative evidence on long‐term trends and inequalities in child mortality.

The findings have several important implications for policy and practice. First, the consistently higher mortality risks among children in poor households and those with less‐educated mothers highlight the urgent need for targeted interventions focused on poverty alleviation and education. This includes expanding access to maternal and child healthcare services and investing in female education. Second, the increased risks associated with early childbearing and short birth intervals underscore the importance of strengthening adolescent reproductive health programs and family planning services, particularly those that encourage delayed marriage and proper birth spacing. Moreover, the persistence of regional disparities, especially in areas such as Sylhet and Rangpur, calls for geographically tailored strategies that improve healthcare infrastructure, resource allocation, and context‐specific interventions in high‐risk regions. Finally, although overall progress is commendable, the uneven distribution of these gains underscores the need for a stronger focus on equity in health policy to ensure that vulnerable populations are not overlooked.

## Author Contributions

Md. Nafiul Islam, Sumaiya Abedin, and Md Ismail Tareque conceived the study and contributed to its investigation. The methodology was developed by Md. Nafiul Islam, Sumaiya Abedin, Ahbab Mohammad Fazle Rabbi, and Md Ismail Tareque, whereas validation was carried out by Md. Nafiul Islam, Sumaiya Abedin, and Ahbab Mohammad Fazle Rabbi. Formal analysis was performed by Md. Nafiul Islam and Ahbab Mohammad Fazle Rabbi, and data curation was undertaken by Md. Nafiul Islam and Md Ismail Tareque. Visualizations were prepared by Md. Nafiul Islam, Ahbab Mohammad Fazle Rabbi, and Md Ismail Tareque, whereas software development was contributed by Ahbab Mohammad Fazle Rabbi. Project administration was managed by Sumaiya Abedin and Md Ismail Tareque. Sumaiya Abedin, Ahbab Mohammad Fazle Rabbi, and Md Ismail Tareque provided supervision and resources for the study. The original draft of the manuscript was prepared by Md. Nafiul Islam, Sumaiya Abedin, Ahbab Mohammad Fazle Rabbi, and Md Ismail Tareque. Manuscript review and editing were carried out by Md. Nafiul Islam, Ahbab Mohammad Fazle Rabbi, and Md Ismail Tareque. All authors revised and proofread the manuscript for intellectual content and gave consent for the publication of the final version.

## Funding

The authors have nothing to report.

## Disclosure

The corresponding author confirms that this manuscript is an honest, accurate, and transparent account of the study being reported; that no important aspects of the study have been omitted; and that any discrepancies from the study as planned (and, if relevant, registered) have been explained.

## Conflicts of Interest

The authors declare no conflicts of interest.

## Data Availability

The data used in this study are free and available for access in www.MeasureDHS.com.
